# 0.9% saline versus Plasma-Lyte as initial fluid in children with diabetic ketoacidosis (SPinK trial): a double-blind randomized controlled trial

**DOI:** 10.1186/s13054-019-2683-3

**Published:** 2020-01-02

**Authors:** Vijai Williams, Muralidharan Jayashree, Karthi Nallasamy, Devi Dayal, Amit Rawat

**Affiliations:** 1 Division of Pediatric Critical Care, Department of Pediatrics, Advanced Pediatrics Centre, Post Graduate Institute of Medical Education & Research, Chandigarh, India; 2 Division of Pediatric Endocrinology, Department of Pediatrics, Advanced Pediatrics Centre, Post Graduate Institute of Medical Education & Research, Chandigarh, India; 3 Division of Pediatric Allergy & Immunology, Department of Pediatrics, Advanced Pediatrics Centre, Post Graduate Institute of Medical Education & Research, Chandigarh, India

**Keywords:** Diabetes, Ketoacidosis, Pediatric, Fluids, Intensive care, Saline, Kidney injury

## Abstract

**Background:**

Acute kidney injury (AKI) is an important complication encountered during the course of diabetic ketoacidosis (DKA). Plasma-Lyte with lower chloride concentration than saline has been shown to be associated with reduced incidence of AKI in adults with septic shock. No study has compared this in DKA.

**Methods:**

This double-blind, parallel-arm, investigator-initiated, randomized controlled trial compared 0.9% saline with Plasma-Lyte-A as initial fluid in pediatric DKA. The study was done in a tertiary care, teaching, and referral hospital in India in children (> 1 month–12 years) with DKA as defined by ISPAD. Children with cerebral edema or known chronic kidney/liver disease or who had received pre-referral fluids and/or insulin were excluded. Sixty-six children were randomized to receive either Plasma-Lyte (*n* = 34) or 0.9% saline (*n* = 32).

**Main outcomes:**

Primary outcome was incidence of new or progressive AKI, defined as a composite outcome of change in creatinine (defined by KDIGO), estimated creatinine clearance (defined by p-RIFLE), and NGAL levels. The secondary outcomes were resolution of AKI, time to resolution of DKA (pH > 7.3, bicarbonate> 15 mEq/L & normal sensorium), change in chloride, pH and bicarbonate levels, proportion of in-hospital all-cause mortality, need for renal replacement therapy (RRT), and length of ICU and hospital stay.

**Results:**

Baseline characteristics were similar in both groups. The incidence of new or progressive AKI was similar in both [Plasma-Lyte 13 (38.2%) versus 0.9% saline 15 (46.9%); adjusted OR 1.22; 95% CI 0.43–3.43, *p* = 0.70]. The median (IQR) time to resolution of DKA in Plasma-Lyte-A and 0.9% saline were 14.5 (12 to 20) and 16 (8 to 20) h respectively. Time to resolution of AKI was similar in both [Plasma-Lyte 22.1 versus 0.9% saline 18.8 h (adjusted HR 1.72; 95% CI 0.83–3.57; *p* = 0.14)]. Length of hospital stay was also similar in both [Plasma-Lyte 9 (8 to 12) versus 0.9% saline 10 (8.25 to 11) days; *p* = 0.39].

**Conclusions:**

The incidence of new or progressive AKI and resolution of AKI were similar in both groups. Plasma-Lyte-A was similar to 0.9% Saline in time to resolution of DKA, need for RRT, mortality, and lengths of PICU and hospital stay.

**Trial registration:**

Clinical trial registry of India, CTRI/2018/05/014042 (ctri.nic.in) (Retrospectively registered).

## Background

Diabetic ketoacidosis (DKA) is being increasingly recognized among children over the last few years amounting to a quarter of all diabetic admissions to a hospital [1, 2]. Fluids and insulin are the cornerstones of DKA management. Protocol-driven fluid resuscitation has been shown to decrease hyperglycemia by improving renal perfusion and decreasing counter regulatory hormones (CRH) [3].

An ideal fluid in this setting would be one that produces a predictable and sustained increase in intravascular volume. Crystalloids possess a chemical composition close to that of extracellular fluids and get completely metabolized and excreted. Understandably they have been the initial fluid of choice for several decades be it in septic shock or DKA. Among the various crystalloids available, like 0.9% saline, Ringer’s Lactate (RL), and Plasma-Lyte, 0.9% saline has the added advantage of being available in various combinations (N/2, N/3, N/5 and DNS with 5% dextrose). Its cost-effectiveness makes it ideal for a resource-limited setting. Despite the aforementioned advantages, there has been a lot of debate around the ideal resuscitation fluid for use in septic shock and DKA [4].

Recently increasing concerns with the use of 0.9% saline mainly attributed to its supra-physiological concentrations of sodium and chloride have emerged. Preclinical and early clinical data suggest that 0.9% saline may have several adverse effects [5, 6]. Some reports have hypothesized a causal link between hyperchloremia and acute kidney injury (AKI) [7]. Studies in critically ill adults have demonstrated higher risk of AKI and need for RRT with chloride liberal fluids [5, 7]. Studies in adults have reported lesser hyperchloremic acidosis and renal injury and thus better outcome with “balanced” chloride restrictive fluids [8–10]. Although initial randomized trials in septic shock comparing Plasma-Lyte and 0.9% saline (SPLIT and SALT trials) did not show any risk reduction in AKI, the later trials with larger sample size (SMART and SALT-ED trials) showed reduction in major adverse kidney events [11–14]. Extrapolating from the findings of septic shock, a similar risk of hyperchloremia and AKI with 0.9% saline (chloride liberal) in DKA is plausible. An Australian study comparing Hartmann’s solution (chloride restrictive) with 0.9% saline in children with DKA found that former was non-inferior with respect to time to resolution of DKA [15].

Pediatric DKA especially in low-middle income countries (LMIC) is associated with higher rate of complications due to poor socioeconomic status, non-compliance to therapy, associated comorbidities like malnutrition, sepsis and inadequate treatment at first contact healthcare facility [16–18]. Uncorrected dehydration increases the risk of AKI in these children. In a retrospective review from our institution, about a third of children with DKA had AKI at admission. Elevated chloride level at 24 h of PICU stay was a significant predictor for progressive AKI suggesting a causal link between hyperchloremia and AKI [19].

It was important for us to look into factors that could mitigate the risk of AKI in our children with DKA. We hypothesized that balanced fluids with reduced chloride content may decrease hyperchloremia and AKI. There is no study till date comparing Plasma-Lyte-A with 0.9% saline as the initial fluid in pediatric DKA.

## Materials and methods

This was a prospective, double-blind, parallel assignment, investigator-initiated randomized controlled trial conducted from August 2017 to December 2018 in the Pediatric Emergency and Intensive Care Units of a large tertiary care, teaching and referral hospital in India. All consecutive children > 1 month to < 12 years who presented to the pediatric emergency room with DKA as defined by the International Society of Pediatric and Adolescent Diabetes (ISPAD-2014) were enrolled into the study [20]. Children with symptomatic cerebral edema (GCS < 8 at presentation) or known chronic kidney disease or liver disease or who had received pre-referral fluids and/or insulin at the time of hospital presentation were excluded. The severity of DKA was classified as mild if pH was between 7.2 and 7.3, moderate if pH was between 7.1 and 7.2, and severe if pH was < 7.1.

### Sample size estimation

Sample size was calculated with AKI as a composite outcome using multivariate Cox Proportional Hazard Analysis. A total of 60 subjects (nearly 30 in each group) were estimated, expecting that 30 out of them meet one or other composite AKI criteria. With an alpha error of 5% and power of 80%, we assumed that the hazards of AKI in Plasma-Lyte will be 0.3 times lower than saline and the hazard ratio is constant throughout the study.

Data was analyzed as per intention-to-treat principle. Unadjusted chi-square test was used to analyze the differences in primary outcome. Absolute and relative risks with 95% confidence intervals were calculated. Survival analysis for time to resolution of AKI and DKA were compared with log-rank test. A Cox proportional hazards regression model was used to evaluate the influence of potential confounders on outcome as age, new onset DKA, and severity of DKA. Quantitative variables with normal and non-normal distribution were expressed as mean (with standard deviation) or median (with inter-quartile range) respectively. Unpaired Student’s *t* test or Wilcoxon rank-sum test was used for intergroup comparisons. General linear model repeated measure ANCOVA was used to compare the trends of continuous variables over time. A *P* value (two-tailed) < 0.05 was taken as significant. IBM SPSS Version 21 and R software were used for data analysis.

### Randomization and blinding

The randomization scheme number was generated by a person not involved in the study using a web-based program for 1:1 allocation (http://www.randomization.com). Patients were randomized into 2 groups 0.9% saline and Plasma-Lyte-A by unstratified, block randomization with variable block sizes. The fluids (Plasma-Lyte-A and 0.9% saline) were purchased from Company Baxter (India) Pvt. Ltd., and the company had no involvement in the study protocol, data collection, or analysis. Both study fluids (Additional file 1: Table S1) were packed in identical 500-ml bags, covered with two opaque plastic silver foil wraps by a nurse not involved in study and sequentially numbered as per allocation sequence. The allocation was known only to one member of the administrative staff not involved in the study. The patient assignment was sequential. Patients and treating physicians were blinded to the treatment. Once patient’s eligibility for enrolment was determined, a study nurse blinded to the fluids was ordered to administer the crystalloid solution as per the randomization scheme.

### Treatment protocol and monitoring

Parents or legal guardians of children who satisfied the eligibility criteria were approached by the investigator for a possible enrolment in the emergency department. Parents were free not to participate or to withdraw from the study at any point of time. Written informed consent was obtained from the parents or next of kin prior to enrolment. All children, irrespective of their enrolment in the study, received standard care as per the unit’s existing protocol (Additional file 2: Figure S1). A basic data form including screening details, demographic data, precipitating factors, presenting complaints, pre-referral treatment, nutritional status, and physical, biochemical and hemodynamic findings were completed.

The enrolled children were managed according to the standard clinical protocol for DKA followed in our unit (Additional file 2: Figure S1). Urinary NGAL estimation was done for the purpose of trial. Eligible children who presented in shock [perfusion abnormalities with or without hypotension (blood pressure < 5th centile for age)], received trial fluid bolus of 20 ml/kg over an hour. In those without shock, the fluid volume was calculated as a sum of deficit (65–100 ml/kg) and maintenance for the next 48 h and administered as an hourly infusion. Insulin was started at 0.05 U/kg/h in all after initial hour of fluid therapy. Neurological status was monitored hourly anticipating cerebral edema. Fluids were changed to 0.45% saline and 5% dextrose once blood glucose fell below 250 mg/dl. In case of persistently high blood glucose, the clinician went through a checklist that included patency of intravenous cannula, insulin preparation and its shelf life, and appropriateness of dilution before increasing insulin to 0.1 U/kg/h.

### Implementation of study protocol

The protocol was discussed in multiple sessions with the resident doctors and nurses posted in PICU and Emergency units on the first day of every month throughout the duration of the study. The investigator visited the Emergency and PICU at least twice a day and as and when needed to ensure strict implementation of protocol. Phone number of the investigator was made available in both the units. Posters of protocol design were displayed on the notice boards of the Emergency and PICU. Weekly checks on protocol adherence were carried out by the co-investigators.

Clinical data (respiratory rate, pulse, capillary refill, blood pressure, hydration status, fluid intake, urine output) were continuously recorded, and the values were entered in a pre-designed monitoring sheet. Blood glucose (capillary or venous) was checked every hour and blood gas every 4 h. Urea, creatinine, and electrolytes were measured every 4–8 hourly. We used the enzymatic method of creatinine estimation to prevent interference with non-creatinine products.

For KDIGO staging, if pre-admission creatinine values were available, either the single value or the least value (in case of multiple values) during the previous 3 months was taken as baseline value. If baseline creatinine was unavailable, then a GFR of 127 ml/min and 103 ml/min were assumed for children above 1 year and below 1 year respectively to calculate creatinine using Schwartz formula (Additional file 1: Appendix).

The need for RRT was assessed daily. In addition to renal failure-related data points, the duration of mechanical ventilation (MV), length of ICU and hospital stays from time of study enrolment, and in-hospital mortality were recorded.

### NGAL estimation

The concentration of NGAL was estimated with the commercial HumanLipocalin-2/ NGAL ELISA kit (BioVendor Laboratory Medicina, Czech Republic) in a sandwich enzyme immunoassay as per manufacturer instructions. Limit of NGAL detection was 0.02 ng/ml with inter-assay coefficient of variance (CV) of 7.8% and intra-assay CV of 9.7–9.8%.

### Study outcomes

The primary outcome was incidence of new onset or progressive AKI defined as ONE of the following composite outcomes: change in serum creatinine or urine output as per KDIGO classification OR, change in GFR as calculated using Schwartz formula OR, and change in urinary NGAL. The individual outcomes were divided into quartiles, and the worst quartile was used to define AKI. The standard definitions of individual outcomes are provided in Additional file 1: Appendix. Analysis was done separately for absolute levels and their worst quartiles. The secondary outcomes were rate of resolution of AKI, time to resolution of DKA (pH > 7.3, bicarbonate > 15 mEq/L and normal sensorium), change in chloride, pH and bicarbonate levels (baseline, 24 h), proportion of in-hospital all-cause mortality, proportion of children requiring renal replacement therapy (RRT), length of ICU and hospital stay.

### Follow-up

Patients were followed up till discharge from PICU or ward or death, whichever was earlier. Post discharge, the children were assessed in the PICU and diabetic follow-up clinics. Patients failing to attend day 28 follow-up were contacted through telephone to ascertain the survival status. Renal function tests were carried out wherever needed. A data safety monitoring board supervised the conduct of the trial (Additional file 1: Appendix).

## Results

### Flow of patients

**Fig. 1:**
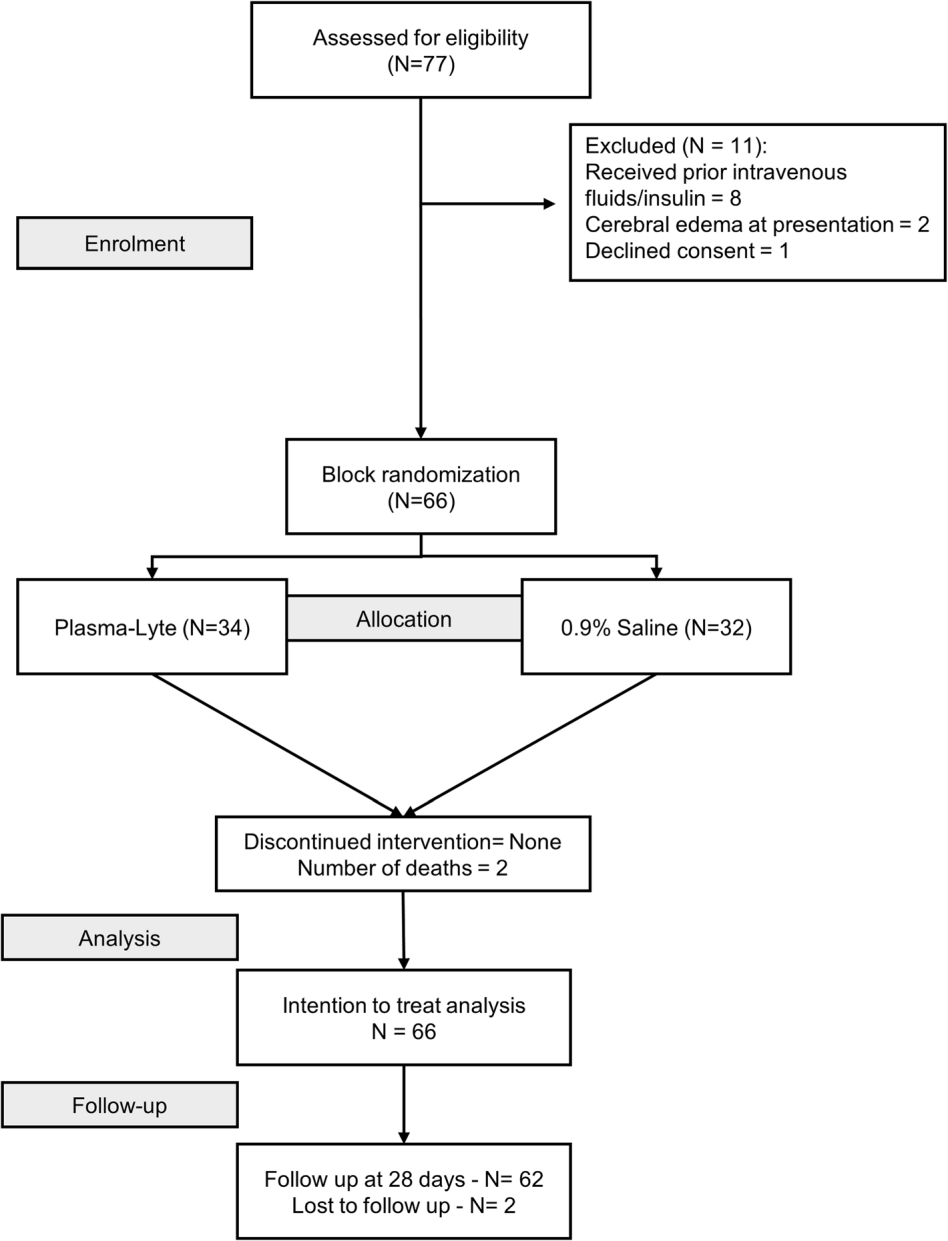
Study flow diagram

A total of 77 episodes of DKA in 75 eligible children were admitted to the Pediatric Emergency during the study period. Of these, 11 were excluded; 8 had received insulin and fluids, 2 had cerebral edema at presentation, and 1 with mild DKA had denied consent. None had chronic kidney disease or liver dysfunction during screening (Fig. 1).

The remaining 66 DKA episodes in 64 children were consecutively included in the study and sequentially randomized to receive either 0.9% saline or Plasma-Lyte-A. The rest of the DKA management conformed to the standard protocol. There was one protocol violation; one child who was started on insulin at 0.1 U/kg/h. No fluid-related violations or contaminations were noted in any of the enrolled children. The fluid-deficit estimation was based on the treating clinician’s assessment. None received sodium bicarbonate. Continuous monitoring including fluid balance and laboratory evaluation were uniform in all children of both groups. All children allocated to each arm were included for analysis.

### Baseline characteristics

The two groups were compared across all baseline characteristics as shown in Tables 1 and 2. Both groups were similar in all aspects, except for more number of new onset DKA in 0.9% saline (75%) as compared to Plasma-Lyte-A (50%) (*p* = 0.03) and a higher median admission blood glucose in Plasma-Lyte-A as compared to 0.9% saline [(488 mg/dl vs 430 mg/dl), *p* = 0.003]. Fluid infusion rate, urine output, and fluid balance were similar in both groups (Additional file 1: Table S2). Thirteen children (9 in Plasma-Lyte-A and 4 in 0.9% saline) required increment of insulin infusion for persisting hyperglycemia.

**Table 1:** Baseline characteristics of study groups

Characteristics	Plasma-Lyte-A (*n* = 34)	0.9% saline (*n* = 32)	*P* value
Age in years	7.8 (4.0, 11.6)	6.6 (2.9, 10.1)	0.305
Male gender, *n* (%)	18 (52.9)	15 (46.9)	0.622
Weight for age (z score)	− 2.9 (− 3.18, − 1.13)	− 1.8 (− 2.77, − 0.67)	0.197
Height for age (*z* score))	− 1.25 (− 2.58, − 0.78)	− 1.32 (− 1.90, − 0.52)	0.441
BMI for age (*z* score)	− 1.79 (− 2.93, − 0.77)	− 1.5 (− 3.0, − 0.77)	0.99
Socioeconomic class*	3 (3, 4)	3 (3, 4)	0.481
New onset DKA, *n* (%)	17 (50)	24 (75)	*0.03*
Duration of diabetes in known type 1 diabetes (months)	26.7 (7.2, 47.8)	15.4 (6.1, 32.2)	0.861
Age of onset of diabetes (months)	79.5 (46.8, 99.7)	79.7 (31.6, 109.6)	0.857
Previous episode of DKA, *n* (%)			
None	23 (67.6)	25 (78.1)	
≤ 2	10 (29.4)	6 (18.8)	
> 2	1 (3.0)	1 (3.1)	
Type of insulin regimen, *n* (%)			
Basal bolus	13 (76.5)	8 (100)	0.134
Spilt mix	4 (23.5)	0 (0)	
Poor compliance to insulin, *n* (%)	13 (76.5)	8 (100)	0.134
Time from onset of symptoms to presentation (days)	4 (2.0, 7.7)	6 (3, 10)	0.23
Severity of DKA, *n* (%)			
Severe	20 (58.8)	20 (62.5)	0.95
Moderate	11 (32.4)	11 (34.4)	0.62
Mild	3 (8.8)	1 (3.1)	0.13
GCS at admission	14 (11, 15)	13 (10, 15)	0.547
Hypotension at admission, *n* (%)	2 (5.9)	2 (6.3)	0.95
Need for PICU, *n* (%)	21 (61.8)	14 (43.8)	0.143

*Kuppusamy Scale 2017, Data expressed as median (IQR)

**Table 2:** Baseline laboratory parameters of study groups

Parameter	Plasma-Lyte -A(*n* = 34)	0.9% saline (*n* = 32)	*P* value
Blood glucose at 0 h (mg/dl)	488 (435, 618)	430 (367, 477)	*0.003*
Blood glucose at 1 h (mg/dl)	470 (389, 579)	393 (326, 456)	*0.004*
Blood Ketones (mmol/L)	5.5 (5.0, 6.6)	5.3 (4.6, 6.3)	0.079
pH	6.98 (6.90, 7.16)	6.99 (6.90, 7.15)	0.974
Bicarbonate (mmol/L)	6.9 (5.8, 9.5)	6.8 (5.4, 8.6)	0.464
pCO_2_ (mm Hg)	20.8 (16.0, 27.6)	19.6 (15.3, 24.8)	0.379
Base deficit (mmol/L)	22.1 (18.5, 25.5)	23.1 (18.4, 25.9)	0.517
Electrolytes			
Sodium (mmol/L)	138.0 (131.5, 143.0)	137.5 (134.2, 141.5)	0.837
Corrected sodium (mmol/L)	143.0 (139, 148.3)	143 (139, 146)	0.639
Chloride (mmol/L)	112.5 (103.7, 117.7)	111 (105.2, 117.7)	0.908
Potassium (mmol/L)	4.1 (3.4, 4.8)	3.8 (3.2, 4.4)	0.264
Phosphate (mg/dl)	2.9 (1.75, 3.2)	2.7 (2.52, 3.5)	0.393
Urea (mg/dl)	31.5 (24.0, 52.0)	32.5 (23.3, 54.8)	0.923
Creatinine (mg/dl)	0.8 (0.6, 1.07)	0.68 (0.5, 1.0)	0.21
Anion gap (mmol/L)	19.8 (16.1, 22.9)	18.9 (15.8, 22.3)	0.419
Osmolality (mmol/kg)	300 (293, 308)	298 (292, 307)	0.369
Lactate (mmol/L)	2 (1.3, 2.5)	2 (1.4, 2.3)	0.903
Urinary NGAL at 0 h (ng/ml)	73.61 (41.7, 95.6)	82.46 (36.1, 92.2)	0.9
Others			
Hemoglobin (g/dl)	11.9 (10.2, 12.9)	11.0 (10.1, 12.3)	0.193
Total leukocyte count (per cu.mm)	13,075 (10,100, 23,775)	13,900 (8895, 21,802)	0.758
HbA1C (%)	12.2 (11.25, 14.0)	12.7 (9.28, 12.75)	0.842
C peptide (ng/ml)	0.16 (0.05, 0.39)	0.33 (0.20, 0.69)	0.016
Vitamin D (ng/ml)	10.48 (4.89, 14.4)	10.27 (3.34, 17.8)	0.881
PTH (pg/ml)	14.55 (8.31, 25.69)	21.6 (13.22, 28.23)	0.275

Data expressed as median (IQR), *p*<0.05 was considered significant

SI conversion factors: To convert glucose to mmol/L, multiply values by 0.055. To convert creatinine to micromol/L, multiply values by 88.4. To convert phosphate to mmol/L, multiply values by 0.323.

### Outcomes

*Primary outcome*: The primary composite outcome when defined by worst quartile showed 28 children with AKI: 13 (38.2%) in Plasma-Lyte and 15 (46.9%) in 0.9% saline. The odds of developing AKI were increased by 22% in 0.9% saline group in comparison to Plasma-Lyte-A (adjusted OR 1.22; 95% CI 0.43–3.43, *p* = 0.70) when adjusted for age, new onset DKA, and severity of DKA at presentation (Additional file 3: Figure S2). However, absolute risk difference between Plasma-Lyte-A and 0.9% saline (i.e., favoring Plasma-Lyte-A) was − 8.64% (with 95% CI 32.4 -15.1%); a 32.4% risk reduction in AKI (lower bound 95% CI value) favoring Plasma-Lyte. We also found that the occurrence of AKI would be reduced under Plasma-Lyte treatment (over and above saline) by 18.4% in the study sample (preventable fraction in exposed) and by 9.5% in target population (preventable fraction by population). Additionally, the number needed to treat (NNT) analysis revealed that for every 12 patients treated with Plasma-Lyte-A (instead of control saline) one additional patient will not develop AKI (the study outcome). New/progressive AKI in first 48 h was similar in both groups when defined with either KDIGO or pRIFLE criteria: 3 (8.8%) in Plasma-Lyte-A and 1 (3.1%) in 0.9% saline group [unadjusted risk ratio 3.0; 95% CI 0.29–30.44), *p* 0.332] (Table 3).

**Table 3:** Outcome measures between study groups

Outcome parameter	Plasma-Lyte-A (*n* = 34)	0.9% saline (*n* = 32)	*P* value	Risk ratio (95% CI)
Primary outcome				
Composite AKI				
AKI based on worst quartiles at 48 h, *n* (%)	13 (38.2)	15 (46.9)	0.491	1.42 (0.53, 3.86)
AKI based on KDIGO and p-RIFLE at 48 h, *n* (%)	3 (8.8)	1 (3.1)	0.332	3.0 (0.29, 30.44)
Individual outcomes of composite AKI				
Creatinine >75th percentile, *n* (%)	6 (17.6)	5 (15.6)	0.837	0.87 (0.22, 3.31)
Urinary NGAL >75th percentile, *n* (%)	9 (27.3)	7 (21.9)	0.629	0.75 (0.23, 2.38)
GFR < 25th percentile, *n* (%)	7 (20.6)	10 (31.2)	0.341	1.73 (0.56, 5.59)
Secondary outcomes				
Reduction in AKI at various time points				
0 h, *n* (%)	21 (61.8)	15 (46.9)	0.225	1.82 (0.69, 4.88)
24 h, *n* (%)	4 (11.8)	3 (9.4)	0.753	1.28 (0.26, 6.27)
48 h, *n* (%)	3 (8.8)	1 (3.1)	0.332	3.0 (0.29, 30.44)
Time to resolution of AKI (hours)	22.1 (13.8, 30.5)	18.8 (15.1, 24.3)	0.494	0.98 (0.94, 1.03)
Time to reach DKA endpoint (hours)	14.5 (12, 20)	16 (8, 20)	0.472	0.96 (0.86, 1.13)
Chloride elevation first 4 h	3.5 (0.75, 7.25)	4.0 (1, 10)	0.403	
Chloride elevation first 8 h	4.0 (0.50, 8.50)	6.0 (1, 9.5)	0.608	
Need for RRT, *n* (%)	2 (5.9)	0 (0)	0.164	
Need for ventilation, *n* (%)	2 (5.9)	1 (3.1)	0.591	
Mortality in hospital, *n* (%)	2 (5.9)	0(0)	0.164	
Length of ICU stay (hours)	48 (48, 60)	47 (24, 54)	0.276	
Length of Hospital stay (days)	9 (8, 12)	10.0 (8.25, 11)	0.396	
28-day survival, n (%)	31 (94.1)	31 (100)	0.164	

Data expressed as median (IQR)

*Secondary outcomes* were also compared between both groups (Table 3). The overall mortality of the study cohort was 3% (*n* = 2); there was no mortality difference between groups (*p* = 0.94). One child succumbed to fungal sepsis, shock, and MODS at 72 h of PICU stay. This child had received trial fluid as first hour bolus and subsequently for 4 h before switch to 0.45% saline with 5% dextrose. The second child had AKI stage 2 at admission and developed cerebral edema at 6 h at which point the trial fluid was stopped and restricted fluid regimen was initiated. She succumbed at 56 h of PICU stay to progressive MODS.

### Resolution of AKI

Serial assessment every 8 hourly showed a decreasing trend of AKI stage. At admission, the incidence of AKI (defined with either KDIGO or pRIFLE criteria) was 21 (61.8%) and 15 (46.9%) in Plasma-Lyte-A and 0.9% saline group respectively. With initiation of fluids, AKI at 24 and 48 h was reduced to 4 (11.8%) and 3 (8.8%) respectively in Plasma-Lyte-A and 3 (9.4%) and 1 (3.1%) respectively in 0.9% saline group.

Although the rate of resolution of AKI was early in 0.9% saline group when compared to Plasma-Lyte-A group (18 h versus 22 h), this difference was not statistically significant (adjusted HR 1.72; 95% CI 0.83–3.57; *p* = 0.14). Time to resolution of AKI as defined by worst quartile comparing both fluid groups was also non-significant (Additional file 4: Figure S3).

### Time to resolution of DKA

**Fig. 2:**
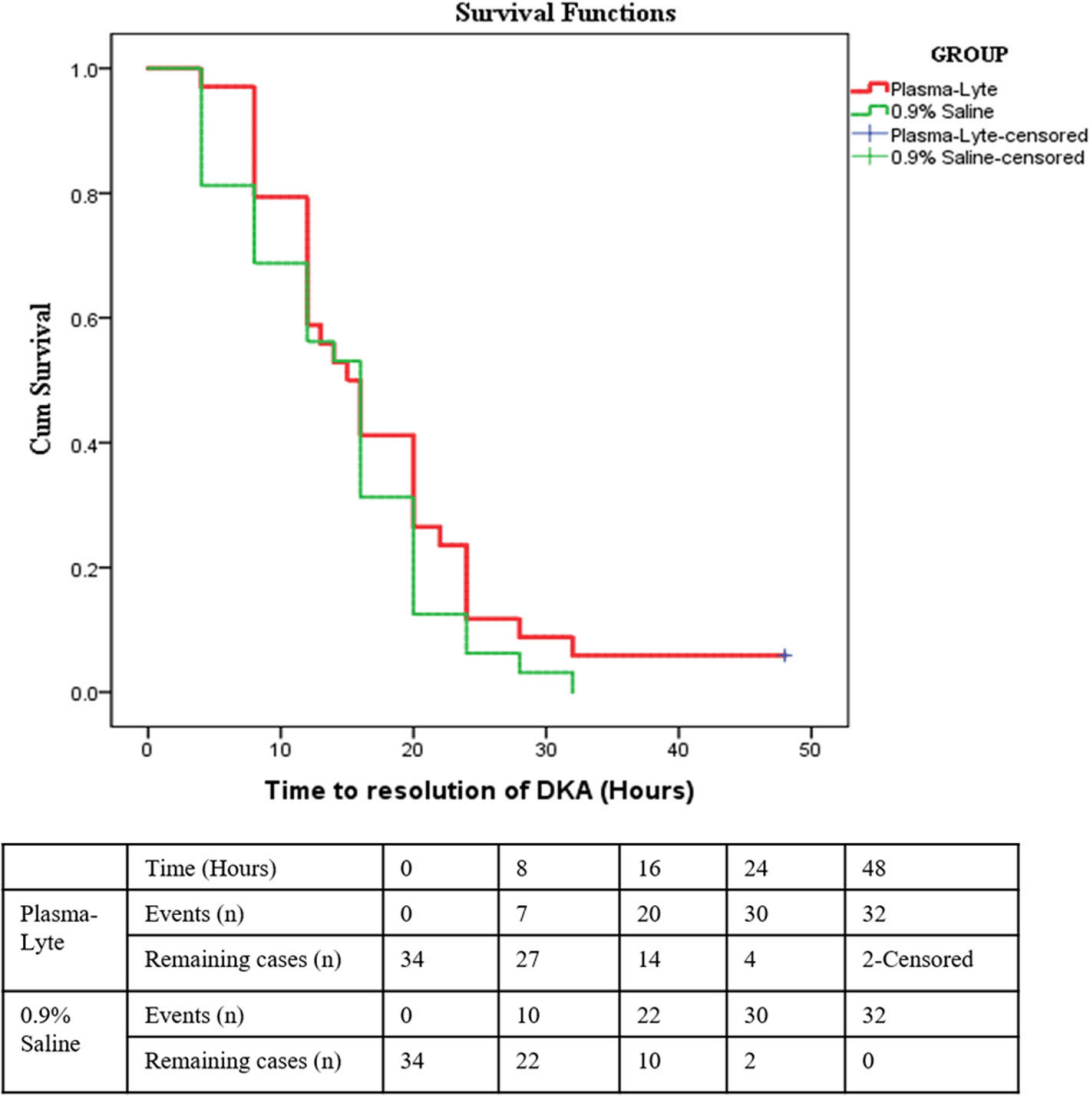
Survival curve–time to resolution of DKA between study groups

The median (IQR) time to resolution of DKA in Plasma-Lyte-A and 0.9% saline were 14.5 (12 to 20) h and 16 (8 to 20) h respectively. A similar trend was observed in the time to event analysis as shown in Kaplan-Meier curve (Fig. 2).

The baseline median (IQR) chloride was high in both groups: 112.5 (103.7 to 117.7) mEq/L in Plasma-Lyte-A and 111 (105.2 to 117.7) mEq/L in 0.9% saline group. Although there was elevation in chloride levels in initial hours of therapy, the difference remained statistically insignificant. The improvement in pH, closure of anion gap, rate of bicarbonate and sodium increase, and decrease in calculated effective osmolality during therapy between both groups were similar (Additional file 5: Figure S4 and Additional file 6: Figure S5).

Complications seen were hypokalemia in 22 (33%), hypoglycemia in 5 (7.5%), and cerebral edema in 1 (1.5%). Nineteen (28%) children required increment of fluid dextrose concentration to 7.5% to allow for continued insulin use. The incidence of complications in both arms was similar (Additional file 1: Table S3). A subgroup analysis comparing different fluid volumes showed that children who received < 75 ml/kg had faster resolution of DKA as compared to those who received more > 75 ml/kg fluids. We found that the former group had a significantly higher proportion of mild and moderate DKA as compared to later group (*p* = 0.001). Subgroup analysis done assuming a lower mean of normal GFR at 90 ml/min as baseline also did not reveal any significant difference in AKI between study groups (Additional file 1: Table S4). Median (IQR) length of PICU stay [Plasma-Lyte-A 48 (48 to 60) vs 0.9% saline 47 (24 to 54) h; *p* = 0.276] and length of hospital stay [Plasma-Lyte-A 9 (8 to 12) vs 0.9% saline 10 (8.25 to 11) days; *p* = 0.396] were similar in both groups. Of the 64 children discharged, 2 were lost to follow-up at 28 days’ post discharge.

## Discussion

We found that 0.9% saline and Plasma-Lyte-A as initial fluid in pediatric DKA were similar with respect to incidence of new or progressive AKI, time to resolution of DKA, change in chloride levels, need for RRT, and duration of ICU or hospital stay.

Our cohort comprised of a higher proportion of children with severe DKA. The severity, complications, morbidity, and mortality associated with DKA in LMIC are higher than that reported from the West. The putative reasons include lower socioeconomic status, missed or delayed diagnosis in new onset DM, poor adherence to therapy in known TIDM, and inadequate pre-referral optimization with fluids and insulin [16, 17]. These are further compounded by lack of follow-up and continuum of care among known TIDM as reflected by the high HbA1c levels in the present study indicating poor glycemic control. Together, these factors result in severe metabolic decompensation at the time of presentation.

Prolonged uncorrected dehydration/hypovolemia and inadequate fluids in such children aggravates the risk for AKI [16]. This is reflected in the fact that more than half (54.5%) had AKI (stages 2 and 3) at admission itself. Similar observations were reported in a retrospective study on DKA from our institution where the baseline incidence of AKI was 35% [19]. The increased incidence of AKI in the current study compared to our previous published data was possibly related to a more stringent definition of AKI used in this study. Incidence of AKI in critically ill DKA varies from 50% in adults [21] to 64.2–85% in children [22]. Also, it has been seen that cautious rehydration protocols adopted to prevent brain injury in DKA had in turn increased AKI [23, 24].

In the current study, AKI resolving in most over 24–48 h with appropriate fluid therapy suggests that baseline AKI was secondary to dehydration and hypovolemia. It is important to prevent other triggers that can aggravate AKI. This is the basis of interest around chloride liberal versus restrictive fluids in both DKA and septic shock. A total of 4 studies have been published till date on balanced fluids in DKA (3 adult and 1 pediatric) [15, 25–27]. The only pediatric RCT by Yung et al. comparing Hartmann solution with 0.9% saline did not show any difference in time to resolution of DKA or complications [15].

Mahler et al. in their randomized trial comparing Plasma-Lyte-A with 0.9% saline showed lower mean chloride elevation (8 mmol/L vs 16.5 mmol/L) and higher mean bicarbonate (20 mmol/L vs 17 mmol/L) with Plasma-Lyte in adults with moderate to severe DKA. However, whether prevention of hyperchloremic acidosis translated into clinically significant outcomes was not analyzed [25]. Hyperchloremic acidosis has been described more prominently in patients who received either rapid boluses or large volume of fluids during resuscitation for septic shock [5, 7]. The gradual deficit correction and infrequent fluid bolus requirement in the current study could possibly explain the absence of significant hyperchloremia. The median fluid received was 64 ml/kg and 68 ml/kg in Plasma-Lyte A and 0.9% saline groups respectively which is less than the usual deficit volumes of 100 ml/kg required in adults with DKA. The cause for hyperchloremia therefore seems more of a normal physiological response to loss of bicarbonate over chloride with improved renal perfusion than due to fluid type or volume. Dose-related effect on hyperchloremia however needs further exploration.

The causal link between hyperchloremia and AKI is yet to be conclusively established. Observational studies have shown conflicting results on AKI and high chloride content [8, 28, 29]. However, randomized controlled trials in adults have failed to demonstrate a clear cut association [11, 30]. In the current study, the slight increase in chloride levels in both arms did not translate into increase AKI. In light of the limited statistical power, our finding of reduction in AKI with Plasma-Lyte-A is hypothesis generating and should be the subject of future investigations.

We did not find any significant difference in time to resolution of acidosis in either groups similar to the study comparing Hartmann solution and saline [15]. The time to resolution of acidosis in the current study was higher than the reported mean time of 8 to 14 h but similar to a previously published report from our institution [31]. The higher proportion of severe DKA seems a likely reason for this.

### Strengths and limitations

This is the first study of its kind in pediatric DKA comparing Plasma-Lyte A with 0.9% saline as initial fluid in a cohort with severe metabolic decompensation and high risk for AKI. It was an investigator-initiated double-blind randomized controlled trial. Patient screening and enrolment were stringent. No contamination of groups occurred. There was near complete adherence to fluid protocol, meticulous monitoring, and no missing values requiring assumptions. This study is also one of the first that has used NGAL as a component of the composite definition for AKI. The study however has certain limitations. The study is still underpowered as the occurrence of primary outcome in both groups, despite being a composite variable, was few. Achieving a larger sample size in a single-center study is difficult and requires a multicentric study.

## Conclusion

This preliminary study comparing 0.9% *S*aline versus *P*lasma-Lyte A in children with diabetic *k*etoacidosis (SPinK) as initial fluid showed a high incidence of AKI in pediatric DKA. The incidence of new or progressive AKI and resolution of AKI were similar in both groups. Plasma-Lyte-A was similar to 0.9% Saline in reduction of AKI, resolution of DKA, need for RRT, mortality, and duration of PICU or hospital stay.

## Supplementary information

**Additional file 1:Table S1.** Composition of different crystalloids. **Table S2.** Comparison of fluid balance of study groups. **Table S3.** Complications of DKA between study groups. **Table S4.** Incidence of AKI if GFR assumed as 90 ml/min. **Appendix.** Clinical outcome definitions. Details of Data Safety Monitoring Board (DSMB).

**Additional file 2: Figure S1.** Study work pathway.

**Additional file 3: Figure S2.** Hazard ratio for developing AKI.

**Additional file 4: Figure S3.** Survival curve- Time to resolution of AKI between study groups.

**Additional file 5: Figure S4.** Trends of pH, bicarbonate and anion gap.

**Additional file 6: Figure S5:** Trends of serum chloride, corrected sodium and effective osmolality.

## Data Availability

The datasets used and/or analyzed during the current study are available from the corresponding author on reasonable request.
