# Supplementary material for: 0.9% saline versus Plasma-Lyte as initial fluid in children with diabetic ketoacidosis (SPinK trial): a double-blind randomized controlled trial
Source: Crit Care. 2020 Jan 2;24:1. doi: 10.1186/s13054-019-2683-3 (PMC6939333; doi:10.1186/s13054-019-2683-3)
Supplement: Supplementary file 1 — Additional file 1:Table S1. Composition of different crystalloids. Table S2. Comparison of fluid balance of study groups. Table S3. Complications of DKA between study groups. Table S4. Incidence of AKI if GFR assumed as 90 ml/min. Appendix. Clinical outcome definitions. Details of Data Safety Monitoring Board (DSMB). [file 13054_2019_2683_MOESM1_ESM.docx]

**Table S1. Composition of different crystalloids**

| **Parameter of fluid** | **Plasma** | **Study fluids** | | **Other crystalloids** | | |
| --- | --- | --- | --- | --- | --- | --- |
|  |  | **Plasma-Lyte A** | **Saline (0.9%)** | **Plasma-Lyte 148** | **Ringer’s Lactate** | **Hartmann’s solution** |
| Osmolality (mOsm/L) | 275-295 | 294 | 308 | 295 | 280 | 279 |
| pH | 7.35-7.45 | 7.4 | 4.5-7.0 | 4.0-8.0 | 6.0-8.0 | 5.0-7.0 |
| Sodium (mmol/L) | 135-145 | 140 | 154 | 140 | 130 | 131 |
| Potassium (mmol/L) | 3.5-5.0 | 5 | - | 5 | 4.0 | 5.0 |
| Chloride (mmol/L) | 95-110 | 98 | 154 | 98 | 109 | 111 |
| Calcium (mmol/L) | 2.2-2.6 | 0 | - | 0 | 1.4 | 2.0 |
| Magnesium (mmol/L) | 0.8-1.2 | 3.0 | - | 1.5 | 0 | 0 |
| Bicarbonate (mmol/L) | 23-28 | 0 | 0 | 0 | 0 | 0 |
| Acetate (mmol/L) | 0.02-0.2 | 27 | - | 27 | 0 | 0 |
| Gluconate (mmol/L) | 0 | 23 | - | 23 | 0 | 0 |
| Lactate (mmol/L) | 1-2 | 0 | - | 0 | 28 | 29 |
| SID | 42 | 50 | 0 | 50 | 27 | 27 |
| Na: Cl ratio | 1.21-1.54:1 | 1.43:1 | 1:1 | 1.43:1 | 1.19:1 | 1.18:1 |

*SID-Strong ion difference

**Table S2. Comparison of fluid balance of study groups**

| **Variable** | **Plasma-Lyte (N=34)** | **0.9% saline (N=32)** | **P value** |
| --- | --- | --- | --- |
| Total fluid, ml | 1200 (760,1785) | 1190 (810,1857) | 0.763 |
| Total fluid, ml/kg | 64 (48,98) | 68 (41,102) | 0.888 |
| Study fluid, ml | 531 (358,790) | 490 (308,684) | 0.595 |
| Study fluid, ml/kg | 30 (20,37) | 27 (18, 43) | 0.724 |
| Other fluids, ml | 699 (275, 946) | 712 (1097) | 0.827 |
| Other fluids, ml/kg | 36 (19,61) | 41 (17,63) | 0.923 |
| Study fluid duration, hours | 6 (4.75, 8.0) | 6 (4,8) | 0.335 |
| Fluid overload % (FO%) | 2.54 (14.7,41.6) | 2.65 (11.0, 40.4) | 0.763 |
| Blood glucose fall during initial hour of hydration before starting insulin | 33.5 (-3.0,93.7) | 30 (3.0,59.50) | 0.476 |
| Need for insulin increment, n(%) | 9 (26.5) | 4 (12.5) | 0.154 |

*Data expressed as Median (IQR)

**Table S3. Complications of DKA between study groups**

| **Events** | **Plasma-Lyte (n=34)** | **0.9% saline (n=32)** | **P value** | **Adjusted odds ratio (95% CI)** |
| --- | --- | --- | --- | --- |
| Hypokalemia | 9 (26.5) | 13 (40.6) | 0.22 | 1.90 (0.67,5.37) |
| Hypoglycemia | 2 (5.9) | 3 (9.4) | 0.59 | 1.65 (0.26, 10.6) |
| Children requiring more than 7.5%D | 10 (29.4) | 9 (28.1) | 0.91 | 0.94 (0.32,2.73) |
| Cerebral edema | 1 (2.9) | 0 (0) | 0.328 | 0.97 (0.91,103) |

**Table S4. Incidence of AKI if GFR assumed as 90ml/min**

| **Time since randomisation** | **Plasma-Lyte (n=34)** | **0.9% saline (n=32)** | **p value** | **Risk ratio (95% CI)** |
| --- | --- | --- | --- | --- |
| 0 hours, n(%) | 10 (29.4) | 7 (21.9) | 0.48 | 1.49 (0.48, 4.54) |
| 24 hours, n(%) | 3 (8.8) | 0 (0) | 0.24 | 0.9 (0.29, 1.02) |
| 48 hours, n(%) | 2 (5.9) | 0 (0) | 0.49 | 0.9 (0.88,1.02) |

**Appendix**

**Clinical outcome definitions:**

AKI definition as per **KDIGO criteria** as shown below

| **Stage** | **Serum creatinine** | **Urine output** |
| --- | --- | --- |
| 1 | 1.5 – 1.9 times baseline or ≥0.3 mg/dl increase | <0.5 ml/kg/hr for 6-12 hours |
| 2 | 2.0-2.9 times baseline | <0.5 ml/kg/hr for ≥12 hours |
| 3 | ≥3 times baseline or increase in serum creatinine to ≥4 mg/dl or initiation of RRT or decrease in GFR <35ml/min/1.73 m2 | <0.3 ml/kg/hr for ≥24 hours or anuria for ≥12 hours |

AKI as defined by decline in GFR based on modification of **pRIFLE** criteria

| **Stage** | **pRIFLE** | **Estimated Creatinine clearance (eCrCl)** |
| --- | --- | --- |
| 1 | Risk | Decrease by 25% |
| 2 | Injury | Decrease by 50% |
| 3 | Failure | Decrease by 75% or less than 35 ml/min/1.73m^2^ |

Estimated Creatinine clearance (eCrCl) or Glomerular Filtration Rate was defined by Bedside Schwartz equation as below:

eCrCl (ml/min) = 0.413 x (Height in cm/Serum creatinine in mg/dl)

**NGAL:** Since the cut off for NGAL as a marker for AKI still remains to be validated in children, we defined AKI based on change in NGAL from baseline to 24 hours.

| AKI | Normal/ elevated baseline value which increased subsequently  Elevated baseline value which continued to remain elevated |
| --- | --- |
| No AKI | Baseline low value that remained static  Elevated baseline that fell to a lower value |

**D.** Stages 0 and 1 (mild) were further categorized as **NO AKI** group and Stages 2 (moderate) and 3 (severe) were classified as **AKI** group

For children in whom baseline creatinine or nadir value of serum creatinine over last 3 months were unavailable, we resorted to using Schwartz formula for creatinine calculation with assumption of GFR of 120 ml/min as normal. A subgroup analysis with assumption of GFR of 90ml/min as normal was also done. (Table S5) Modification of Diet in Renal Disease (MDRD) equation (back‐estimation formula) was another option, however since MDRD is not validated in children we chose the Schwartz equation.

**Details of Data Safety Monitoring Board (DSMB):**

The members of the DSMB were as follows:

 Prof. Savita Kumari (Chair & HOD Internal Medicine)

 Dr. Rama Walia (Member, Endocrinologist)

 Prof. Arun Bansal (Member, Pediatric Intensive care)

 Dr. Ashish Kakkar (Member, Pharmacology and Statistics)

 Prof. M Jayashree (Guide)

 Prof. Devidayal (Co Guide & Head, Pediatric Endocrinology)

We did not conduct interim analysis. The committee was convened at the end of enrollment and deliberated on the adverse events associated with fluid use in DKA.
