# Supplementary figures and images for: 0.9% saline versus Plasma-Lyte as initial fluid in children with diabetic ketoacidosis (SPinK trial): a double-blind randomized controlled trial
Source: Crit Care. 2020 Jan 2;24:1. doi: 10.1186/s13054-019-2683-3 (PMC6939333; doi:10.1186/s13054-019-2683-3)

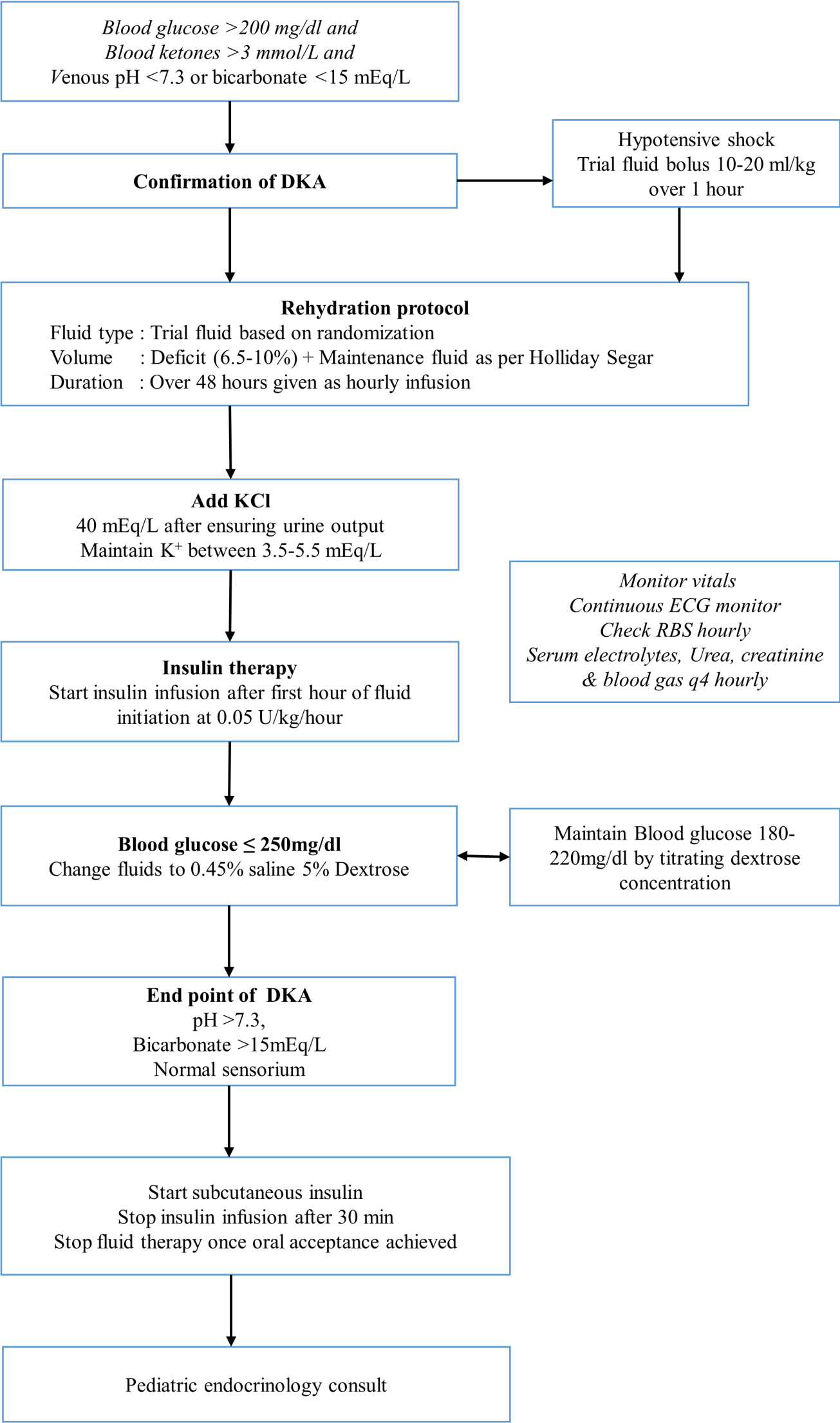

Supplement: Supplementary file 2 — Additional file 2: Figure S1. Study work pathway. [file 13054_2019_2683_MOESM2_ESM.pdf]

Survival Functions

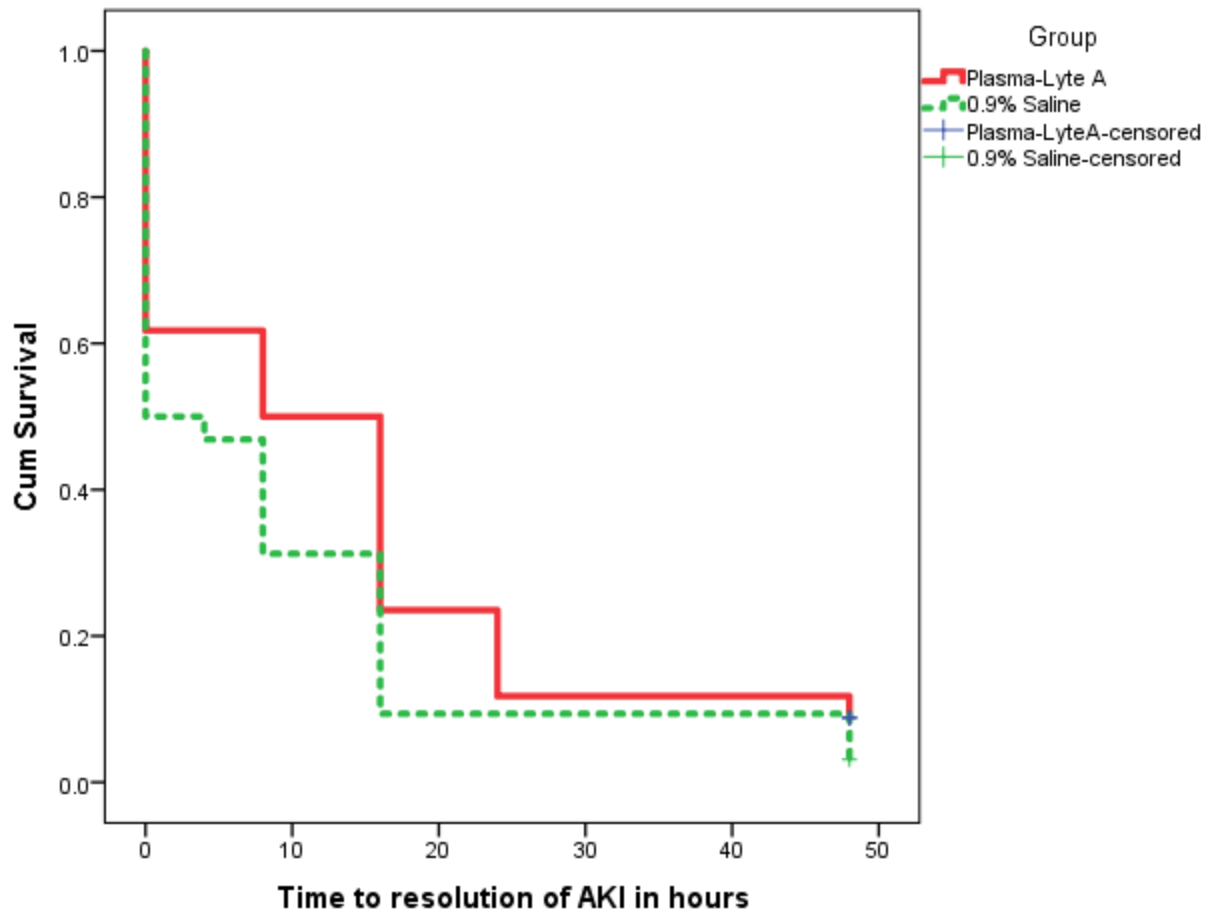

Supplement: Supplementary file 4 — Additional file 4: Figure S3. Survival curve- Time to resolution of AKI between study groups. [file 13054_2019_2683_MOESM4_ESM.pdf]

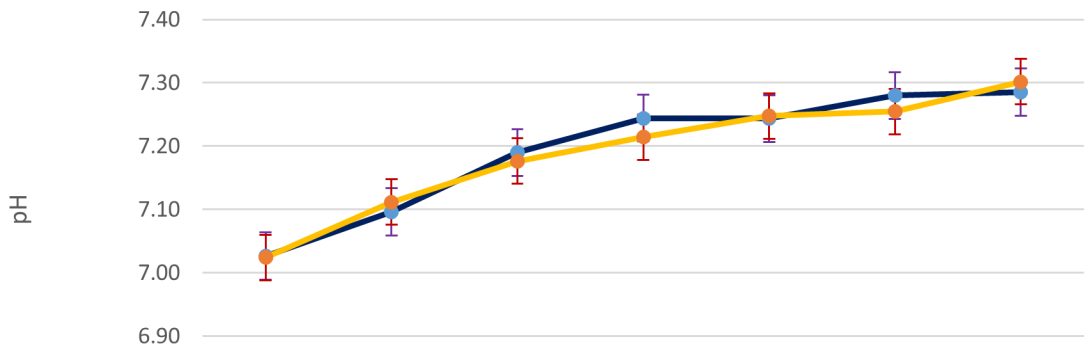

|             | 0    | 4    | 8    | 12   | 16   | 20   | 24   |
|-------------|------|------|------|------|------|------|------|
| Plasma-Lyte | 7.03 | 7.10 | 7.19 | 7.24 | 7.24 | 7.28 | 7.29 |
| 0.9% Saline | 7.02 | 7.11 | 7.18 | 7.21 | 7.25 | 7.25 | 7.30 |

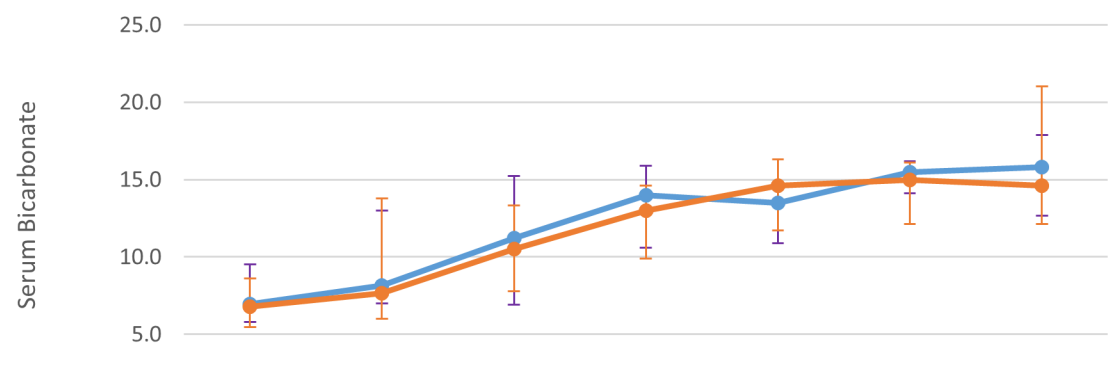

|             | 0   | 4   | 8    | 12   | 16   | 20   | 24   |
|-------------|-----|-----|------|------|------|------|------|
| Plasma-Lyte | 7.0 | 8.2 | 11.2 | 14.0 | 13.5 | 15.5 | 15.8 |
| 0.9% Saline | 6.8 | 7.7 | 10.5 | 13.0 | 14.6 | 15.0 | 14.6 |

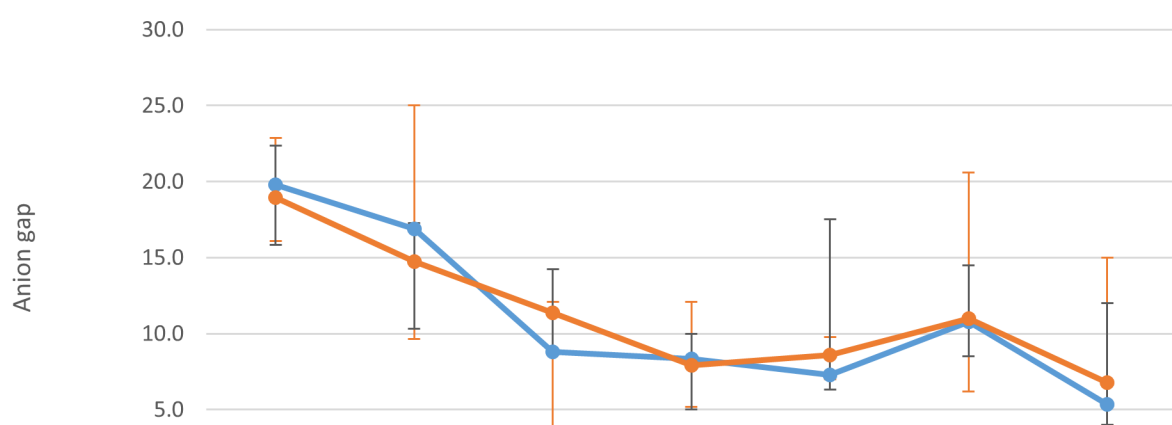

|             | 0    | 4    | 8    | 12  | 16  | 20   | 24  |
|-------------|------|------|------|-----|-----|------|-----|
| Plasma-Lyte | 19.8 | 16.9 | 8.8  | 8.4 | 7.3 | 10.8 | 5.4 |
| 0.9% Saline | 19.0 | 14.8 | 11.4 | 7.9 | 8.6 | 11.0 | 6.8 |

Time

Supplement: Supplementary file 5 — Additional file 5: Figure S4. Trends of pH, bicarbonate and anion gap. [file 13054_2019_2683_MOESM5_ESM.pdf]

Serum Chloride

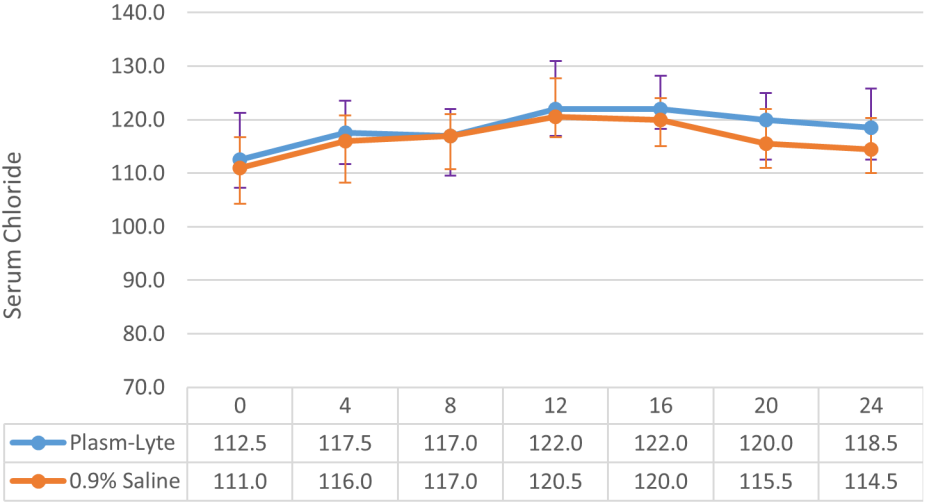

Corrected sodium

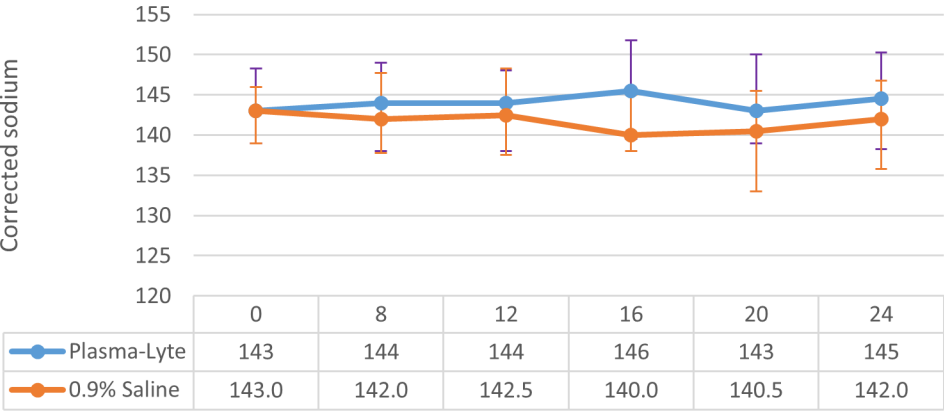

Effective osmolality

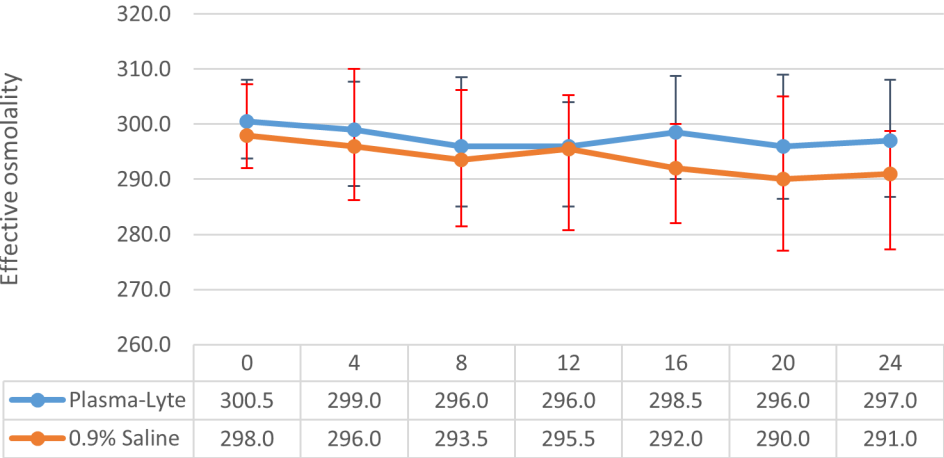

Time

Supplement: Supplementary file 6 — Additional file 6: Figure S5: Trends of serum chloride, corrected sodium and effective osmolality. [file 13054_2019_2683_MOESM6_ESM.pdf]
