# Supplementary material for: 0.9% saline versus Plasma-Lyte as initial fluid in children with diabetic ketoacidosis (SPinK trial): a double-blind randomized controlled trial
Source: Crit Care. 2020 Jan 2;24:1. doi: 10.1186/s13054-019-2683-3 (PMC6939333; doi:10.1186/s13054-019-2683-3)
Supplement: Supplementary file 3 — Additional file 3: Figure S2. Hazard ratio for developing AKI. [file 13054_2019_2683_MOESM3_ESM.pdf]

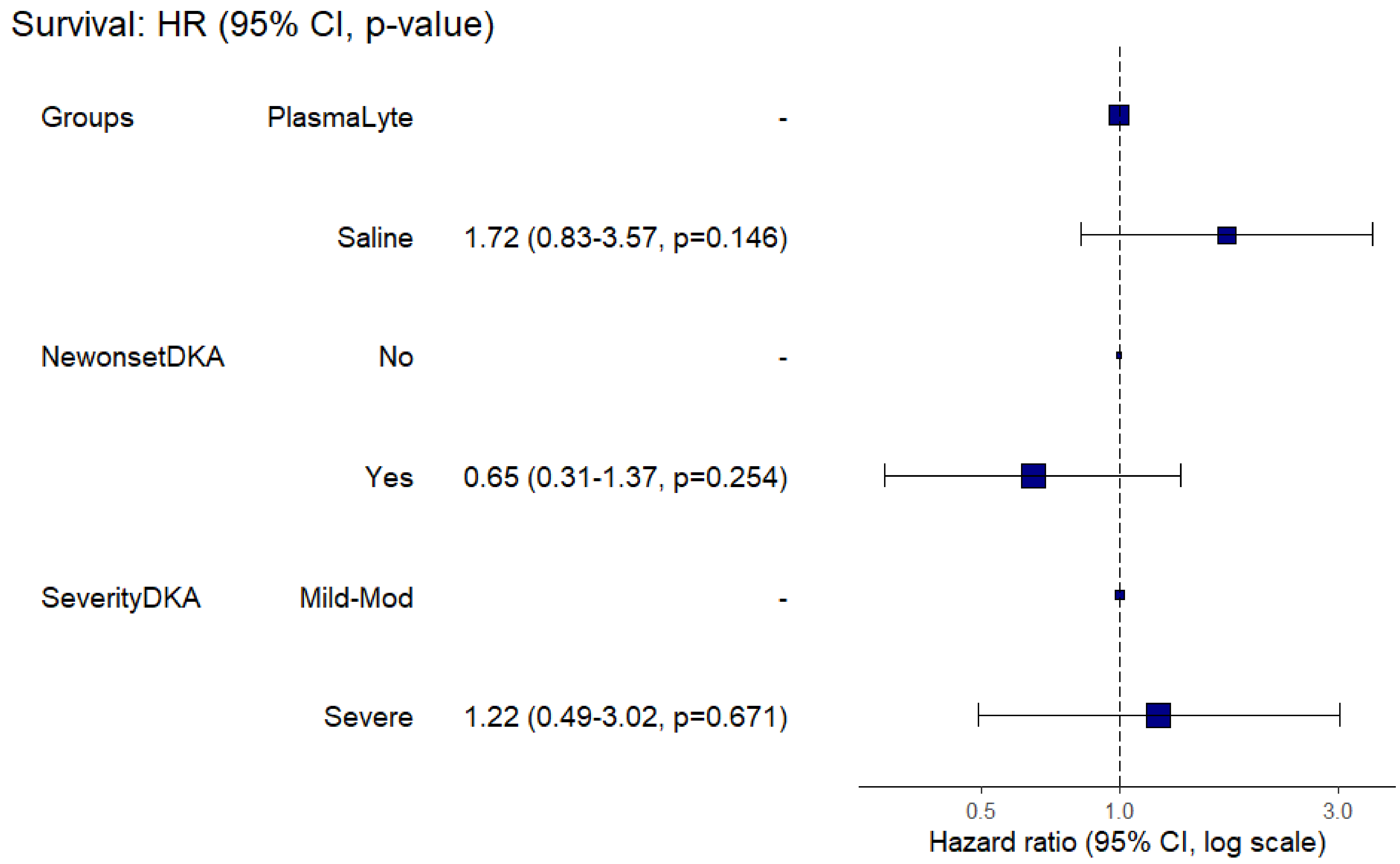
Survival: HR (95% CI, p-value)
Groups
PlasmaLyte
-
Saline
1.72 (0.83-3.57, p=0.146)
NewonsetDKA
No
-
Yes
0.65 (0.31-1.37, p=0.254)
SeverityDKA
Mild-Mod
-
Severe
1.22 (0.49-3.02, p=0.671)
0.5
1.0
3.0
Hazard ratio (95% CI, log scale)
